# A Single-Center, Observational Study of 607 Children and Young People Presenting With Differences of Sex Development (DSD)

**DOI:** 10.1210/jendso/bvac165

**Published:** 2022-10-28

**Authors:** Elim Man, Imran Mushtaq, Angela Barnicoat, Polly Carmichael, Claire R Hughes, Kate Davies, Helen Aitkenhead, Rakesh Amin, Charles R Buchanan, Abraham Cherian, Nikola J Costa, Sarah M Creighton, Patrick G Duffy, Emma Hewson, Peter C Hindmarsh, Louisa C Monzani, Catherine J Peters, Philip G Ransley, Naima Smeulders, Helen A Spoudeas, Dan Wood, Ieuan A Hughes, Harshini Katugampola, Caroline E Brain, Mehul T Dattani, John C Achermann

**Affiliations:** Genetics & Genomic Medicine Research and Teaching Department, UCL Great Ormond Street Institute of Child Health, University College London, London WC1N 1EH, UK; Department of Endocrinology, Great Ormond Street Hospital NHS Foundation Trust, London WC1N 3JH, UK; Department of Paediatrics & Adolescent Medicine, Hong Kong Children's Hospital, Hong Kong SAR, People’s Republic of China; Department of Urology, Great Ormond Street Hospital for Children, London WC1N 3JH, UK; Department of Clinical Genetics, Great Ormond Street Hospital NHS Foundation Trust, London WC1N 3JH, UK; Department of Clinical Psychology, Great Ormond Street Hospital NHS Foundation Trust, London WC1N 3JH, UK; Gender Identity Development Service, Tavistock and Portman NHS Foundation Trust, London NW3 5BA, UK; Department of Endocrinology, Great Ormond Street Hospital NHS Foundation Trust, London WC1N 3JH, UK; Centre for Endocrinology, William Harvey Research Institute, Queen Mary University of London, London EC1M 6BQ, UK; Department of Endocrinology, Great Ormond Street Hospital NHS Foundation Trust, London WC1N 3JH, UK; Institute of Health and Social Care, London South Bank University, London SE1 0AA, UK; Department of Chemical Pathology, Great Ormond Street Hospital NHS Foundation Trust, London WC1N 3JH, UK; Department of Endocrinology, Great Ormond Street Hospital NHS Foundation Trust, London WC1N 3JH, UK; Department of Child Health, King's College Hospital NHS Foundation Trust, London SE5 9RS, UK; Department of Urology, Great Ormond Street Hospital for Children, London WC1N 3JH, UK; Department of Chemical Pathology, Great Ormond Street Hospital NHS Foundation Trust, London WC1N 3JH, UK; Institute for Women's Health, University College London Hospitals NHS Foundation Trust, London NW1 2BU, UK; Department of Urology, Great Ormond Street Hospital for Children, London WC1N 3JH, UK; Department of Clinical Psychology, Great Ormond Street Hospital NHS Foundation Trust, London WC1N 3JH, UK; Department of Endocrinology, Great Ormond Street Hospital NHS Foundation Trust, London WC1N 3JH, UK; Department of Paediatrics, University College London Hospitals NHS Foundation Trust, London NW1 2BU, UK; Department of Clinical Psychology, Great Ormond Street Hospital NHS Foundation Trust, London WC1N 3JH, UK; Department of Endocrinology, Great Ormond Street Hospital NHS Foundation Trust, London WC1N 3JH, UK; Department of Urology, Great Ormond Street Hospital for Children, London WC1N 3JH, UK; Department of Urology, Great Ormond Street Hospital for Children, London WC1N 3JH, UK; Genetics & Genomic Medicine Research and Teaching Department, UCL Great Ormond Street Institute of Child Health, University College London, London WC1N 1EH, UK; Department of Endocrinology, Great Ormond Street Hospital NHS Foundation Trust, London WC1N 3JH, UK; Department of Urology, Great Ormond Street Hospital for Children, London WC1N 3JH, UK; Department of Urology, University College London Hospitals NHS Foundation Trust, London NW1 2BU, UK; Department of Urology, Children's Hospital Colorado and University of Colorado, Aurora, Colorado 80045, USA; Department of Paediatrics, University of Cambridge, Cambridge CB2 0QQ, UK; Department of Endocrinology, Great Ormond Street Hospital NHS Foundation Trust, London WC1N 3JH, UK; Department of Endocrinology, Great Ormond Street Hospital NHS Foundation Trust, London WC1N 3JH, UK; Genetics & Genomic Medicine Research and Teaching Department, UCL Great Ormond Street Institute of Child Health, University College London, London WC1N 1EH, UK; Department of Endocrinology, Great Ormond Street Hospital NHS Foundation Trust, London WC1N 3JH, UK; Genetics & Genomic Medicine Research and Teaching Department, UCL Great Ormond Street Institute of Child Health, University College London, London WC1N 1EH, UK; Department of Endocrinology, Great Ormond Street Hospital NHS Foundation Trust, London WC1N 3JH, UK

**Keywords:** DSD, sex development, ambiguous genitalia, hypospadias, androgen insensitivity, testicular dysgenesis, congenital adrenal hyperplasia

## Abstract

**Context:**

Differences of sex development (DSD) represent a wide range of conditions presenting at different ages to various health professionals. Establishing a diagnosis, supporting the family, and developing a management plan are important.

**Objective:**

We aimed to better understand the presentation and prevalence of pediatric DSD.

**Methods:**

A retrospective, observational cohort study was undertaken in a single tertiary pediatric center of all children and young people (CYP) referred to a DSD multidisciplinary team over 25 years (1995-2019). In total, 607 CYP (520 regional referrals) were included. Data were analyzed for diagnosis, sex-assignment, age and mode of presentation, additional phenotypic features, mortality, and approximate point prevalence.

**Results:**

Among the 3 major DSD categories, sex chromosome DSD was diagnosed in 11.2% (68/607) (most commonly 45,X/46,XY mosaicism), 46,XY DSD in 61.1% (371/607) (multiple diagnoses often with associated features), while 46,XX DSD occurred in 27.7% (168/607) (often 21-hydroxylase deficiency). Most children (80.1%) presented as neonates, usually with atypical genitalia, adrenal insufficiency, undescended testes or hernias. Those presenting later had diverse features. Rarely, the diagnosis was made antenatally (3.8%, n = 23) or following incidental karyotyping/family history (n = 14). Mortality was surprisingly high in 46,XY children, usually due to complex associated features (46,XY girls, 8.3%; 46,XY boys, 2.7%). The approximate point prevalence of neonatal referrals for investigation of DSD was 1 in 6347 births, and 1 in 5101 overall throughout childhood.

**Conclusion:**

DSD represent a diverse range of conditions that can present at different ages. Pathways for expert diagnosis and management are important to optimize care.

Differences of sex development (DSD) (also known as disorders of sex development, variations in sex characteristics, or sometimes intersex) represent a wide range of conditions that can be diagnosed at several different stages of life and that can first present to health professionals in diverse disciplines [1–3].

The most common presentation is in the neonatal period, when a baby is born with atypical (“ambiguous”) genitalia and it is not immediately clear whether the child is a boy or a girl. In other situations, DSD can be diagnosed following a mismatch between prenatal karyotype and phenotype; due to associated features in childhood (eg, renal, hernias); during adolescence with absent puberty, virilization, estrogenization or primary amenorrhea; or sometimes in adulthood with infertility or as an incidental finding.

DSD was originally defined as situations in which “chromosomal, gonadal or anatomical sex is atypical” [4]. Three major categories are usually considered: sex chromosome DSD (SCDSD, where there is an imbalance in sex chromosome complement), 46,XY DSD, and 46,XX DSD. However, these are only general categories and many specific conditions exist within them. Reaching a specific diagnosis is crucial for initial management, support, and education, as well as for planning long-term care through adolescence and into adulthood [3, 5, 6].

Despite progress at many levels, the debate about what constitutes “DSD” continues and the incidence of different DSD diagnoses and their relative prevalence is not well established. More common genital variations such as distal hypospadias may affect up to 1 in 250 to 300 boys [7], whereas the prevalence of “DSD” requiring further investigation is often cited as approximately 1 in 5000 children, but robust data are limited [8–10]. One of the most important DSD-related conditions, classic congenital adrenal hyperplasia (CAH) (mostly 21-hydroxylase deficiency), affects approximately 1 in 13 000 to 18 000 of all children in most countries, and approximately 1 in 30 000 to 45 000 of all newborns will be a 46,XX infant with atypical genitalia due to CAH [11]. In contrast, 46,XY gonadal dysgenesis (sometimes called “Swyer syndrome”) and complete androgen insensitivity syndrome (CAIS) are less common, and often present during the teenage years [12]. Important global differences in the incidence of different conditions also exist.

To address some of these questions and to obtain a better understanding of DSD demographics, we present an overview of 25 years’ experience of DSD from a single-center pediatric multidisciplinary team (MDT). We provide insight into the referral patterns, range of presentations and diagnoses, associated features, and approximate prevalence of conditions.

## Materials and Methods

### Cohort and Setting

The study included all children and young people (CYP) discussed at the monthly DSD MDT meeting at Great Ormond Street Hospital (GOSH) NHS Foundation Trust between January 1, 1995 and December 31, 2019. This forum brings together pediatric specialists in psychology, urology, endocrinology, and allied disciplines (including clinical genetics, biochemistry, gynecology, and nursing) to review new referrals, and also provides input at times when follow-up discussions are required. Services for young people first presenting in the mid-teenage years (aged > 13 years) are generally provided by University College London Hospitals and these data are not included here.

**Figure 1.**
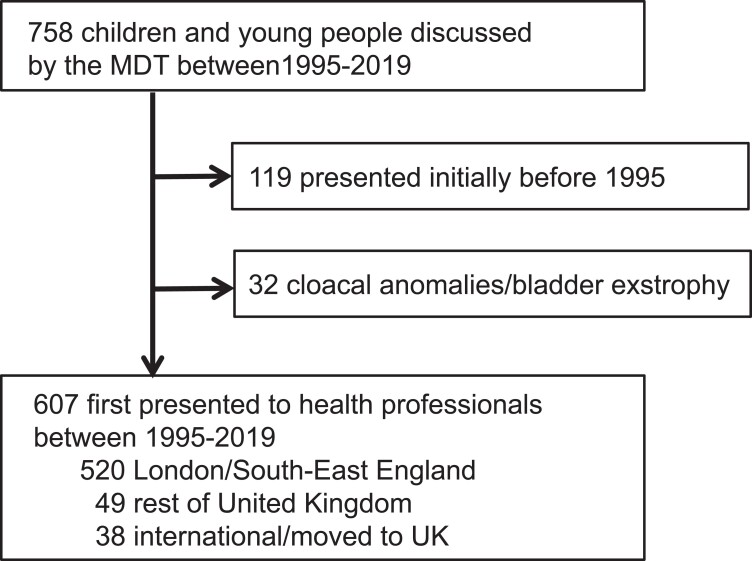
Overview of the study cohort.

A total of 758 case records were reviewed (Fig. 1). Children were excluded if their initial presentation was before 1995 but they were under long-term follow-up (n = 119), as this subgroup was biased because it included children with more complex diagnoses rather than reflecting the relative proportion of new presentations accurately. Children with cloacal anomalies or bladder exstrophy (n = 32) were also excluded as an independent specialist service for these conditions was established during the study period (see Fig. 1). Therefore, the final study cohort included 607 CYP who first presented to health professionals between 1995 and 2019. Of these, 520 CYP were “regional” referrals from London and the South-East of England, 49 were from the rest of the United Kingdom, and 38 were international referrals or CYP whose families relocated to the United Kingdom after the initial diagnosis had been made (see Fig. 1, see also Supplemental Fig. 1) [13].

### Data Acquired

Individual records were retrospectively evaluated by 2 observers (E.M., J.C.A., with input also from C.R.H. and K.D.). A diagnostic category was assigned to each child based on available data and the template of the Chicago Consensus Meeting [4].

Key data obtained from records included karyotype, biochemical and molecular genetic diagnosis, sex assignment, age and mode of first presentation, additional clinical features or syndromes, and mortality.

### Ethical Review

This study was approved as a clinical service evaluation by the GOSH Audit office (1706: Evaluation of clinical pathways for children with disorders of sex development) under NHS research guidelines.

### Statistical Analysis

Data trends over time were analyzed using 3-point moving averages and categorical variables were compared using chi-square analysis (IBM SPSS Statistics 27, IBM Corp). Two-tailed *P* values of less than .05 are considered statistically significant.

### Reporting Principles

As this is a retrospective, cross-sectional observational study, data are presented using STROBE (Strengthening the Reporting of Observational Studies in Epidemiology) principles.

## Results

### Overview of the Cohort

**Figure 2.**
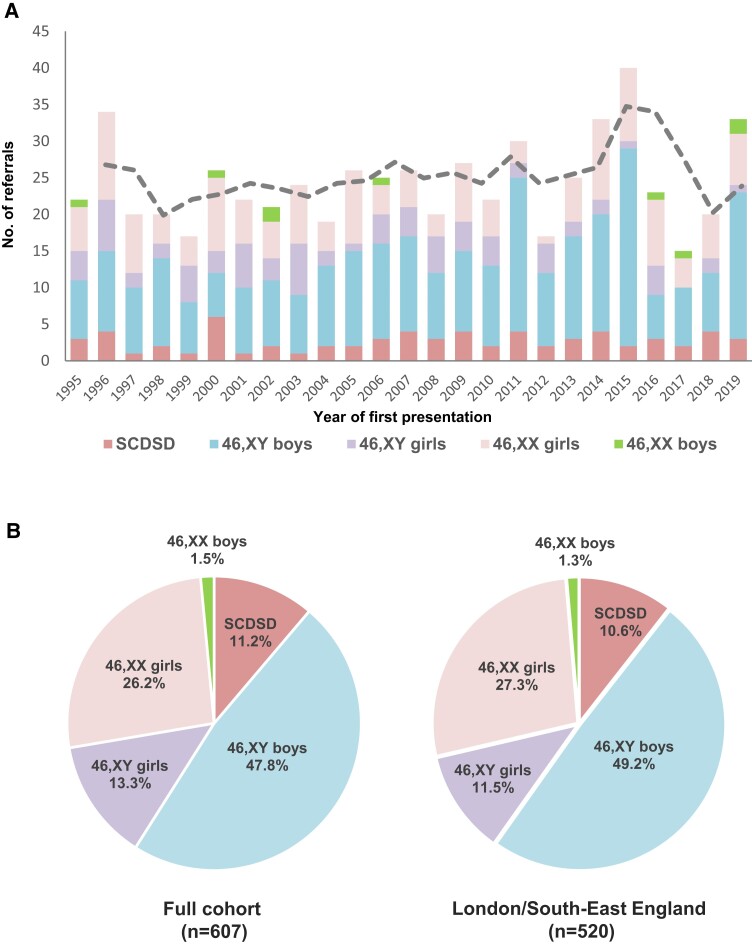
A, Number of children and young people (CYP) presenting to health professionals and being referred to the multidisciplinary team each year over 25 years (3-point moving average shown by dashed line). B, Major differences in sex development (DSD) category and sex assignment for the total cohort (n = 607) (left panel) and for regional referrals from London and the South-East of England (n = 520) (right panel). SCDSD, sex chromosome DSD.

The median number of new children presenting to the MDT each year was 23 (range, 15-40) (total n = 607) (Fig. 2A). There were no statistically significant changes over time (*R* = 0.20; *P* = .33). SCDSD accounted for 11.2% (68/607) of individuals, 46,XY DSD was found in 61.1% (371/607), and 46,XX DSD occurred in 27.7% (168/607) (Fig. 2B). Overall there was discordance between karyotype and sex in 14.8% of individuals for the study cohort as a whole (13.3% 46,XY children brought up as girls, hereafter termed “*46,XY girls*”; 1.5% 46,XX children brought up as boys, hereafter termed “*46,XX boys*”) (see Fig. 2B). The percentage of 46,XY girls was slightly lower (11.5%, 60/520) when regional referrals from just London and the South-East of England were considered (see Fig. 2B). Of note, many children had a phenotype consistent with the sex they were brought up in and presented for other reasons (eg, a phenotypic girl with complete androgen insensitivity syndrome and 46,XY karyotype presenting with an inguinal hernia). Less frequently, a child had atypical genitalia and a decision was made by the parents together with the MDT to bring the child up in a sex different from their karyotype (see “46,XY girls”).

### Categories of Differences of Sex Development

An overview of the diagnoses in each major category of DSD is shown in Table 1 and Supplementary Figs. 2 to 4 [13].

**Table 1.** Overview of study cohort of children referred

Diagnosis	n	% of cohort (n = 607)	Diagnosis	n	% of cohort (n = 607)
**Sex chromosome DSD (SCDSD)**	**68**	**11**.**2**	**46,XY DSD**	**371**	**61**.**1**
45,X and variants	2	0.3	Hypothalamic-pituitary disorders	15	2.4
45,X/46,XY and variants	56	9.2	*HH with hypopituitarism*	*6*	*0*.*9*
47,XXY	5	0.8	*HH—isolated*	*9*	*1*.*4*
46,XX/46,XY chimerism	4	0.7	Disorders of gonad development	85	14.0
Other (47,XXX)	1	0.2	*GD—unknown cause*	*9*	*1*.*4*
			*GD—syndrome*	*5*	*0*.*8*
			*Partial GD—unknown cause*	*20*	*3*.*3*
**46,XX DSD**	**168**	**27**.**7**	*Partial GD—syndrome*	*6*	*1*.*0*
Disorders of gonad development	13	2.2	*SRY*	*2*	*0*.*3*
*(Ovo)testicular DSD*	*13*	*2*.*2*	*SOX9*	*1*	*0*.*2*
Disorders of androgen excess	102	16.8	*NR5A1 (SF-1)*	*8*	*1*.*3*
*CAH—3β-HSD type 2 deficiency*	*3*	*0*.*5*	*WT1*	*12*	*2*.*0*
*CAH—21-hydroxylase deficiency*	*92*	*15*.*2*	*GATA4*	*1*	*0*.*2*
*CAH—11β-hydroxylase deficiency*	*5*	*0*.*8*	*Testis regression/anorchia*	*21*	*3*.*5*
*Aromatase deficiency*	*1*	*0*.*2*	Disorders of androgen synthesis	43	7.08
*Adrenocortical tumor*	*1*	*0*.*2*	*Smith-Lemli-Opitz syndrome*	*1*	*0*.*2*
Other conditions	42	6.9	*Leydig cell hypoplasia*	*2*	*0*.*3*
*MRKH syndrome*	*8*	*1*.*3*	*STAR*	*2*	*0*.*3*
*Syndromes with genital anomalies*	*3*	*0*.*5*	*CAH—3β-HSD type 2 deficiency*	*2*	*0*.*3*
*Ovarian anomaly—isolated*	*5*	*0*.*8*	*CAH—17α-hydroxylase/17,20-lyase def.*	*3*	*0*.*5*
*Vaginal/labial anomaly—isolated*	*2*	*0*.*3*	*CAH—P450 oxidoreductase def.*	*1*	*0*.*2*
*Clitoral anomaly—isolated*	*7*	*1*.*2*	*17β-HSD type 3 deficiency*	*16*	*2*.*5*
*Clitoromegaly with prematurity*	*17*	*2*.*8*	*5α-reductase type 2 deficiency*	*16*	*2*.*5*
Within “normal” limits	11	1.8	Disorders of androgen action	31	5.1
			*Complete AIS*	*17*	*2*.*8*
			*Partial AIS*	*8*	*1*.*3*
			*Partial AIS-like*	*6*	1.0
			Persistent müllerian duct syndrome	7	1.2
			Penoscrotal hypospadias	125	20.6
			*Penoscrotal hypospadias—isolated*	*33*	*5*.*4*
			*Penoscrotal hypospadias—FGR*	*63*	*10*.*4*
			*Penoscrotal hypospadias—cardiac*	*3*	*0*.*5*
			*Penoscrotal hypospadias—renal*	*3*	*0*.*5*
			*Penoscrotal hypospadias—complex*	*23*	*3*.*8*
			Other disorders	64	10.5
			*Penile anomaly—complex*	*23*	*3*.*8*
			*Undescended testes—isolated*	*6*	*1*.*0*
			*Testicular anomaly—isolated*	*1*	*1*.*2*
			*Testicular anomaly—syndrome*	*4*	*0*.*7*
			*Syndromes with genital anomalies*	*30*	*4*.*9*
			Within “normal” limits	1	0.2

Other conditions are shown by gene name. For further information on syndromic associations see Supplementary Table S1 and Supplementary Fig. S3C [13]. “Within ‘normal’ limits” most often included 46,XX girls referred for assessment who had labial adhesions, perineal edema, or excess clitoral skin. The 3 main categories of DSD are shown in bold. Key diagnostic groups are shown in regular text. Sub-diagnoses within these groups are shown in italics.

**Abbreviations:** AIS, androgen insensitivity syndrome; CAH, congenital adrenal hyperplasia; DSD, differences (disorders) of sex development; FGR, fetal growth restriction (also known as IUGR, intrauterine growth restriction); GD, gonadal dysgenesis; HH, hypogonadotropic hypogonadism; HSD, hydroxysteroid dehydrogenase; MRKH, Mayer-Rokitansky-Küster-Hauser syndrome; PMDS, persistent müllerian duct syndrome; SF-1/NR5A1, steroidogenic factor-1; STAR, steroidogenic acute regulatory protein/congenital lipoid adrenal hyperplasia; WT1, Wilms tumor 1.

#### Sex chromosome differences of sex development

Most children presenting with SCDSD had 45,X/46,XY mosaicism or variations of this karyotype (56/68, 82.4%) (see Table 1 and Supplementary Fig. 2) [13]. Within this 45,X/46,XY group, 70% (39/56) of children were brought up as boys and 30% (17/56) were brought up as girls. True 46,XX/46,XY chimerism was very rare, being found in only 4 children in 25 years (5.9% of SCDSD, and 0.7% (4/607) of the total cohort overall). Although some studies consider Turner syndrome/monosomy X within a DSD classification, the vast majority of girls with classic Turner syndrome do not present with DSD and did not get referred through a DSD MDT pathway.

#### 46,XY differences of sex development

The largest and most diagnostically diverse category was 46,XY DSD (see Table 1 and Supplementary Fig. 3) [13]. While there were many specific diagnoses affecting testis development, androgen synthesis, and androgen action, a large number of this cohort were boys with penoscrotal hypospadias without a specific diagnosis (125/371, 33.7%) (see Table 1). Many children in this group had fetal growth restriction (FGR) (sometimes called intrauterine growth restriction) (63/125, 50.4%), additional/complex features (18.4%), or isolated cardiac (2.4%) or renal anomalies (2.4%) (see Supplementary Fig. 3B) [13]. Indeed, in the 46,XY cohort as a whole, 57 of 371 (15.4%) children had a defined genetic syndrome (35 different conditions) and a further 78 of 371 (21.0%) had nonspecific complex phenotypes (which may represent as yet undiagnosed conditions), giving a total of 135 of 371 (36.4%) children with significant additional features (Supplementary Table S1) [13].

#### 46,XX differences of sex development

The most common diagnosis within the 46,XX group was CAH (100/168, 59.5%). Within the CAH group, 21-hydroxylase deficiency (21OHD) was by far the most prevalent condition (92/100, 92.0%) (see Table 1 and Supplementary Fig. S4) [13]. Clitoromegaly associated with prematurity was seen in 10.1% (17/168) children, whereas a small but important group of girls who were referred had variations of anatomy considered to be within the “normal” range (11/168, 6.5%). Ovotesticular or testicular DSD (OTDSD/TDSD) was present in 7.7% children (13/168; 8 raised female, 5 raised male). Of note, OTDSD was more common in 46,XX children from an African/Black British background (5/16 [31.3%]) compared to children from a White background (2/79 [2.5%]; chi-square = 16.08; *P* < .0001). Indeed, OTDSD was at least as common as CAH (21OHD) in 46,XX children of African or Black British background (OTDSD, 5/16 [31.3%] vs 21OHD 3/16 [18.8%]; chi-square = 0.67; *P* = .41). In contrast, CAH (21OHD) was 24-fold more common than OTDSD in 46,XX children of a White background (21OHD 52/79 [65.8%] vs OTDSD 2/79 [2.5%]; chi-square = 70.33; *P* < .0001). Only 11 children with 46,XX DSD had syndromic associations, most often Mayer-Rokitansky-Küster-Hauser (MRKH) syndrome, a form of uterine agenesis (n = 8) (see Supplementary Table S1) [13]. MRKH syndrome does not present with atypical genitalia and is usually diagnosed during the teenage years, but some girls were referred in childhood with hernias containing descended gonads (ovaries), or because of incidental findings of an absent uterus.

### Presentation of Differences of Sex Development

**Figure 3.**
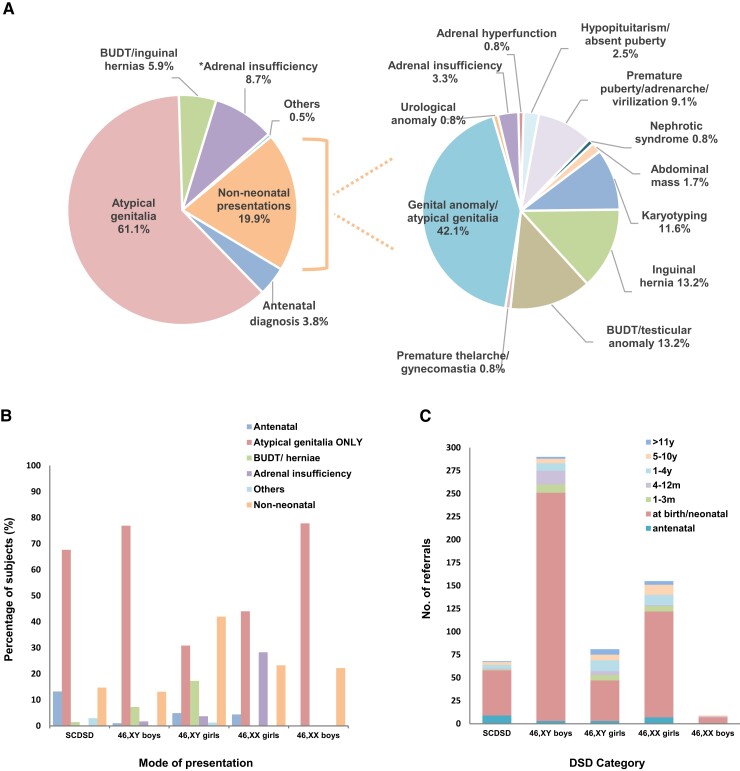
Initial presentation of differences in sex development (DSD). A, Overview of presentation for the total cohort (n = 607) (left panel) and for children presenting outside the neonatal period (n = 121) (right panel). B, Mode of presentation for major DSD categories (n = 607). C, Age at presentation for different major DSD categories (n = 607). *“Adrenal insufficiency” includes 46,XX children with adrenal insufficiency due to congenital adrenal hyperplasia (with atypical genitalia), and two 46,XY girls with congenital lipoid adrenal hyperplasia (STAR). BUDT, bilateral undescended testes; SCDSD, sex chromosome DSD.

Within the entire cohort, most children (486/607, 80.1%) presented during the neonatal period (age < 28 days) usually due to isolated atypical genital appearances (371/486, 76.3% of neonatal referrals), but sometimes also because of undescended testes and hernia(s) (36/486, 7.4%) or adrenal insufficiency (53/486, 10.9%; 48 with atypical genitalia) (Fig. 3A, left panel). An antenatal diagnosis had been made in 3.8% of children in the entire cohort (23/607), 11 of whom had atypical genitalia at birth. Within the London and South-East region, 352 of 520 (67%) patients presented with atypical genitalia.

Those children presenting after the first month (121/607, 19.9%) showed a range of different features (see Fig. 3A, right panel). Genital anomalies were still a surprisingly common reason for presentation, often due to delayed presentation/referral of children with clitoromegaly or a small penis (51/121, 42.1%). Bilateral undescended testes (BUDT)/inguinal hernias were the presenting features in 16 of 121 (13.2%) children, of whom 5 had CAIS (46, XY), 1 had 17β-hydroxysteroid dehydrogenase deficiency type 3 (*HSD17B3*) (46,XY), 1 had a WT1-associated condition (46,XY), 5 had persistent müllerian duct syndrome (PMDS) (46,XY), and 4 had MRKH syndrome (46,XX, inguinal hernias). A diagnosis of DSD after the neonatal period was made in 14 (11.6%) children by karyotyping, either as an incidental finding during genetic testing for other features or because of a known family history of DSD. Other modes of presentation are shown in Fig. 3A.

Presentation with atypical genitalia in the newborn period was the most common presentation in all major subgroups of DSD except 46,XY girls, who were almost as likely to present later in infancy or childhood as in the first month, or who often presented with BUDT/inguinal hernias rather than atypical genitalia (see Fig. 3B and 3C).

### 46,XY Girls (Girls With a Y)

Available data for 46,XY girls were analyzed in more detail, as one of the more challenging situations is when a 46,XY child presents in the newborn period with atypical genitalia and discussions are held with parents as to whether to bring the child up as a girl or boy. We therefore focused on children referred from London and the South East Region of England to obtain a better reflection of UK practice (see Fig. 1 and Supplementary Fig. 1) [13]. Within this group, 46,XY girls represented 11.5% (60/520) of all children (see Fig. 2B).

**Figure 4.**
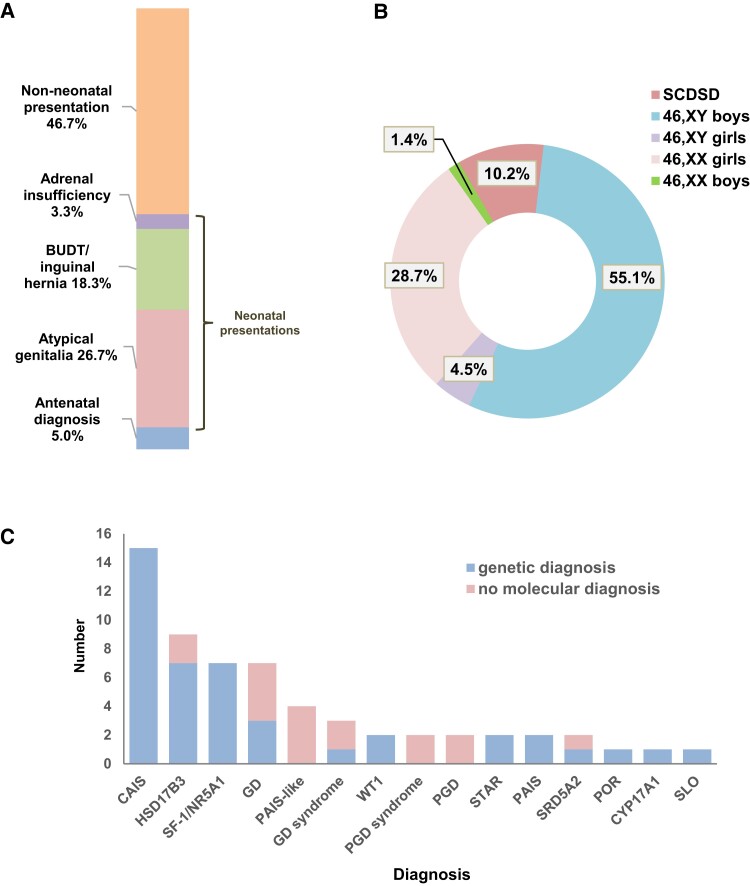
Presentation of 46,XY girls referred from London/South-East England. A, Mode of presentation for 46,XY girls (n = 60). B, Major DSD categories and sex assignment for children presenting with atypical genitalia at birth or in the neonatal period (n = 352). C, Range of genetic diagnoses for 46,XY girls (n = 60). Those children with complete or partial gonadal dysgenesis and additional features are grouped under “GD syndrome” and PGD syndrome,” respectively. “PAIS-like” represents children with clinical/biochemical features of PAIS but in whom no androgen receptor or other pathogenic variant was found yet. BUDT, bilateral undescended testes; CAIS, complete androgen insensitivity syndrome; CYP17A1, combined 17α-hydroxylase/17,20-lyase deficiency; GATA4, GATA-binding protein 4; GD, gonadal dysgenesis; HSD17B3, 17β-hydroxysteroid dehydrogenase type 3 deficiency; PAIS, partial androgen insensitivity syndrome; PGD, partial gonadal dysgenesis; POR, P450 oxidoreductase deficiency; SLO, Smith-Lemli-Opitz syndrome; SRD5A2, 5α-reductase type 2 deficiency; STAR, steroidogenic acute regulatory protein/congenital lipoid adrenal hyperplasia; WT1, Wilms tumor 1.

Although around half of all 46,XY girls presented early in life (29/60, 48.3%, with 3 additional children diagnosed prenatally), only 16 of them (16/60, 26.7%) presented with virilized atypical genitalia (ie, clitoral enlargement, partial labioscrotal fusion) in the newborn period, as opposed to palpable inguinoscrotal or labioscrotal testes but no signs of androgenization (Fig. 4A). This represents 4.5% (16/352) of all “regional” children presenting with atypical genitalia in the newborn period (Fig. 4B) and 3.1% (16/520) of “regional” DSD referrals overall, so this is not a common scenario. Of note, no 46,XY child with partially androgenized genitalia was raised female in the last 5 years of the study.

Genetic testing was offered and a specific genetic diagnosis was available in most children (43/60, 71.7%), with the range of conditions shown in Fig. 4C. A prior family history of DSD was present in 9 of 29 (31.0%) 46,XY girls who presented in the newborn period and a genetic diagnosis was reached in 8 of them.

### 46,XX Boys

Five children from London/South-East England with a 46,XX karyotype and atypical genitalia in the newborn period were brought up as boys (5/352, 1.4% of all newborns with atypical genitalia; 5/520, 0.96% of all “regional” DSD referrals) (see Fig. 4B). Most of these children had OTDSD. An additional two 46,XX boys presented after the first month of life.

### Mortality

Seventeen children died within the follow-up period when records were available (SCDSD, n = 1; 46,XY DSD, n = 16). Median age of death was 5 months (age range, 1 month-9 years). All children had established syndromes (eg, X-linked lissencephaly/*ATRX*, campomelic dysplasia/*SOX9*, Scimitar syndrome), chromosomal deletions or complex multisystem involvement, often associated with fetal growth restriction. Mortality was sometimes linked to neurological/brain anomalies (n = 5), cardiac defects (n = 3), or immune dysfunction and sepsis (n = 3), although children often had complex multiorgan dysfunction. Mortality in the first year of life was higher in 46,XY girls (5/60, 8.3%), compared to 46,XY boys (7/256, 2.7%; chi-square = 4.46; *P* < .05), usually linked to the aforementioned specific syndromes that were associated with severe gonadal dysgenesis. Mortality was substantially higher than national rates of mortality for children within these age ranges (infant mortality rate, 0.37%; child mortality rate (1-15 years), 0.008%; 2019 Office for National Statistics [ONS] England & Wales).

### Incidence of Differences of Sex Development

Based on ONS data for birth rates in England and Wales between 1995 and 2019, and assuming that the “regional” catchment area (London/South-East, Supplementary Fig. S1) [13] represents one-seventh of this population, the number of live births “captured” averaged approximately 106 000 per annum (2 653 000 total births over the study period) [14]. Based on these data, the estimated point prevalence of DSD referrals in the neonatal period (first 28 days) was 1 in 6347 overall (418/2 653 000; 15.8/100 000); 1 in 32 753 for 21OHD (81/2 653 000; 3.1/100 000); 1 in 85 906 for 46,XY girls overall (32/2 653 000; 1.16/100 000); and 1 in 165 812 for 46,XY girls with virilized genitalia (16/2 653 000; 0.6/100 000) (Supplementary Table S2) [13]. The overall prevalence of DSD referrals over the study period was 1 in 5101.

## Discussion

Here we present a large, single-center study of the demographics of DSD, involving more than 600 children over a 25-year period between 1995 and 2019. We used the framework of the Chicago Consensus Meeting to divide children into the 3 broad categories of sex chromosome DSD (SCDSD); 46,XY DSD; and 46,XX DSD, and used available phenotypic, biochemical, and genetic data to make a more specific diagnosis wherever possible.

With this approach, we found that most children referred to specialist services had variations of 46,XY DSD (61.1%), with smaller proportions having 46,XX DSD diagnoses (27.7%), or SCDSD (11.1%). Referral patterns were remarkably stable over this 25-year period, with some year-on-year fluctuations given the small numbers of some categories considered. The predominance of 46,XY DSD has been seen in most other smaller single-center studies to date (Table 2) [15–19]. In contrast, a recent multicenter registry study from the United States has described a higher proportion of children with 46,XX CAH (57%), but this finding may reflect differences in referral pathways and a higher threshold for referring some forms of penoscrotal hypospadias to specialist DSD services [20].

**Table 2.** Overview of studies reporting the relative prevalence of differences of sex development categories

Study author*^a^*	Study period	Country	Study design	Total No.	Inclusion	SCDSD (%)	46,XY DSD (%)	46,XX DSD (%)
This study	1995-2019	UK	Single center	607	DSD MDT forum	11.2	61.1	27.7
Ganie et al [15]	1995-2014	South Africa	Single center	416	DSD diagnosis in an endocrine clinic	9.5	57.5	33*^b^*
De Paula et al [16]	1989-2011	Brazil	Single center	408	Atypical genitalia	8.3	61.3	30.4
Ata et al [17]	2002-2018	Turkey	Single center	289	DSD diagnosis in an endocrine clinic	29	49.5	21.5
Juniarto et al [18]	2004-2010	Indonesia	Single center	286	DSD diagnosis in an endocrine clinic	8.4	68.2	23.4
Heeley et al [19]	1995-2016	USA	Single center	128	Atypical genitalia	11.7	53.1	35.2
Finlayson et al [20]	2013-2017	USA	Multicenter (12 centers)	99	Atypical genitalia (moderate-severe)	9	34	57*^c^*
Rodie et al [10]	2013-2019	Scotland	Multicenter	92	DSD referral at birth	8	70	22

Abbreviations: DSD, differences of sex development; MDT, multidisciplinary team; SCDSD, sex chromosome differences of sex development; UK, United Kingdom; USA, United States of America.

a Studies were included for which karyotype data were available for all 3 diagnostic categories and for which the number of individuals reported was greater than 90.

b Within 46,XX DSD, 60.5% of children had ovotesticular DSD.

c Within 46,XX DSD, 91% of children had 21-hydroxylase deficiency.

Children with sex chromosome variations (SCDSD) were the smallest group within the cohort (11.1%). Here, the diagnosis can usually be made on karyotype alone. Most of these children had 45,X/46,XY mosaicism, or related variations, and 70% were brought up as boys. In fact, based on published antenatal data, more than 90% of children with a 45,X/46,XY karyotype are boys who would not present to health professionals, at least in the early years, so those children with atypical genitalia represent a relatively small subgroup of all children with this karyotype [21, 22]. Given the phenotypic variability and asymmetry of internal and external anatomy often seen in these children, an individualized approach to management is needed [21, 23, 24], taking into account the degree of gonadal compromise, the likelihood of gonadal tumors, and the use of growth hormone to promote growth. Long-term surveillance is also required for potential features associated with Turner syndrome [25]. A similar individualized approach is also required for the small group of children with 46,XX/46.XY chimerism, who can have both ovarian and testicular tissue and who also have variable and often asymmetrical features, although in our series all 4 children with this karyotype were brought up as girls.

The largest and most diverse group was 46,XY DSD (61%). Some of these children had specific diagnoses but many were classified with “penoscrotal hypospadias (PSH). A large subset of these boys had FGR/IUGR and were born preterm. The association of PSH and FGR is well established and can even occur in the smaller of two 46,XY dizygotic twins [26, 27]. Established genetic diagnoses (such as partial androgen insensitivity) are rarely found, but can occur coincidentally [27]. The link between FGR and PSH may reflect an epigenetic phenomenon, a shared placental/genital gene, or a hypothesized interplay between placental insufficiency and fetal androgen synthesis. Furthermore, FGR is a key feature of specific conditions such as MIRAGE syndrome (*SAMD9*) and IMAGe syndrome (*CDKN1C*) [28]. Our study clearly demonstrated that many children (36.4%) with 46,XY DSD phenotypes had complex additional features or a range of defined syndromes; this finding is becoming well established [19, 29]. Indeed, mortality was especially high in the 46,XY children with complex features, especially in the first year of life (> 20 times higher than current national data), reflecting often complex neurodevelopmental issues, cardiac defects, or immune dysfunction. Thus, MDT support, including specialist clinical genetics input, is important for families. Increased availability of clinical genetic testing using whole-genome comparative genomic hybridization arrays, targeted gene panels, or exome sequencing may play a role in defining these associations further in coming years, leading hopefully to more focused management plans [30, 31]. Even in those children without associated features, making a specific diagnosis early on, such as 5α-reductase deficiency, can have substantial implications for management, and genetic analysis has an increasing role to play in this situation as biochemical analysis is unreliable in early life [6].

One important subgroup of 46,XY DSD is those children with a 46,XY karyotype who are brought up as girls. These children represented 11.5% of the entire “regional” cohort, but only 4.5% of newborn babies presenting with virilized atypical genitalia. Of note, some babies with CAIS had atypical appearing genitalia due to the presence of descended labial testes causing swelling, but without evidence of genuine androgenization/virilization. Recent studies have suggested that there may be changes in approaches to sex assignment over time, so that 46,XY children with virilized atypical genitalia are less likely to be brought up as girls than in previous decades [32]. The numbers of children in our study are likely to be too small to draw firm conclusions, although a similar trend was seen. Moreover, a specific genetic diagnosis was reached in most 46,XY girls (73.3%), and higher in those with a family history, which has important implications for targeting support, and planning short- and long-term management [30, 31].

Within the 46,XX category (27.7%) the most important and most common diagnosis is CAH, and the most prevalent form of CAH was 21OHD. CAH should be considered as the diagnosis until proven otherwise in any newborn baby with atypical genitalia and nonpalpable testes, as the child is at risk of adrenal insufficiency and a progressive potentially life-threatening salt-losing state that can develop during the first week of life [33]. Other rare forms of CAH were diagnosed, often in children whose families originate from regions where there are known geographical hotspots (eg, 11β-hydroxylase deficiency in Turkey). Of note was the observation that OTDSD was especially common in 46,XX newborns of African or Black British ancestry. Although rare overall, OTDSD was at least as common as 21OHD in children from this background, in keeping with recent data from several centers in Africa (see Table 2) [15, 34–36]. As the management of OTDSD is markedly different from CAH, this is an important diagnosis to consider. Measuring antimüllerian hormone can be useful to investigate for the presence of testicular tissue, although CAH should always be the working diagnosis until proven otherwise, as it can be life-threatening if not detected and treated appropriately.

Several 46,XX newborns were diagnosed with persistent clitoromegaly associated with prematurity. In some children, the clitoromegaly can persist or is associated with excess clitoral skin, but other signs of early in utero androgen exposure (eg, labial fusion, common urogenital sinus) are not present [37]. The etiology of this condition is not well understood [38]. Furthermore, a small but important group of 46,XX children referred had labial adhesions, excess clitoral skin or genital anatomy within “normal” limits. Labial swelling can occur in some situations (eg, breech deliveries) and there can be individual variability in genital appearances and ancestral differences in clitoral size in different populations [39–42]. Giving reassurance in such situations, after any simple investigations the clinical team feel are necessary, is important.

Establishing the incidence of DSD is challenging, as there are a diverse range of conditions and a spectrum of severity for each of them, and there is ongoing debate about what constitutes “DSD.” Two recent population-based studies in Turkey and Ghana have reported an incidence of atypical genitalia or “DSD” in newborn populations as 1 in 821 (18/14 777) and 1 in 356 (26/9255), respectively [43, 44]. However, these studies included all forms of hypospadias (Turkey) or children with variations of their genitalia outside typical ranges (Ghana). Other studies have taken a national registry approach to look at specific subgroups of DSD. In Denmark, where there is a national cytogenetic registry, publications suggest a peak prevalence of women with a 46,XY karyotype (women with a Y) as 1 in 15 625 women (so potentially 1 in 31 000 of the population overall); CAIS as 1 in 24 390 women; and complete gonadal dysgenesis as 1 in 66 667 women [12]. The peak prevalence of 46,XX men has been reported in Denmark as 1 in 28 571 men (so potentially 1 in 57 000 of the population overall) [45]. In these studies the median age of presentation for CAIS was 7.5 years, that for complete gonadal dysgenesis was 17.0 years, for 46,XX males 17.0 years, and for 46,XX males with testicular DSD 25.4 years. These findings highlight the need for long-term follow-up studies. Only a subgroup of children with these conditions present before the teenage years, which explains the lower prevalence in our data (see Supplementary Table S2) [13], but, as previously stressed, these are extremely important diagnoses to make so that long-term support and management can be appropriately planned.

By considering sequential referrals in a single center over a long time period, we have been able to generate approximate prevalence data for childhood DSD overall (∼ 1 in 5100) and for referrals for further investigation and management in the newborn period (∼ 1 in 6347). These calculations assume that our center captures one-seventh of the population in England and Wales; this, however, may be an underestimate as there are inevitable regional biases in referral patterns. Our center often deals with complex, tertiary referrals, but other centers in the region may see children with DSD and other associated conditions (eg, hypospadias), in which case the true prevalence of DSD would be higher. Previously, Thyen et al [9] have suggested a birth prevalence of 1:5000 in Germany, which is similar to our data. In contrast, Rodie et al [10] have recently reported a birth prevalence of children requiring specialist assessment for atypical genitalia of 1:3318 in Scotland, although 68% of these children were assigned at birth before the results of more detailed investigations, so more complex DSD may have a similar prevalence to in our study. Specifically focusing on CAH, national registry data from the British Paediatric Surveillance unit (August 2007-August 2009) suggested an overall CAH incidence of 1 in 18 000 children (1 in 13 000-15 000 in many countries), and that three-quarters of girls present in the newborn period with genital changes [11]. That would lead to an estimated newborn incidence of virilizing CAH in 46,XX children of around 1 in 48 000 of all children. Our data suggest a CAH prevalence of 1 in 32 753 children overall, similar to the calculated birth prevalence of CAH of 1 in 35 757 in Scotland [10]. Of note, the United Kingdom does not have a newborn screening program for CAH.

As with most single-center studies, this study has limitations in relation to referral biases and the demographic mix of referral sources. London is a large city with families from diverse backgrounds, so our data inevitably reflect that. Referrals of children from outside the United Kingdom or who have relocated here generally involved more “complex” diagnoses, and so we excluded these children for analysis of 46,XY girls and in prevalence estimates, as discussed earlier. Other limitations are the somewhat restricted use of genetics to investigate the potential etiology of conditions such as isolated PSH, and we have not systematically included long-term outcome data, such as gender identity [6]. Alternative approaches to studying rare diseases include national and international registry studies (such as iDSD and iCAH) [46, 47].

This study also highlights the many different ways that children with DSD can present and the central role the MDT has to play [2, 48]. Educating a range of allied professionals in the early approaches to a newborn or child with potential DSD is important, including midwives and nurses, fetal medicine specialists, and general surgeons. Increasing emphasis now focuses on early psychological support for families and children and the key value that support groups play in long-term management throughout childhood, transition during the teenage years, and into long-term adult management pathways [49, 50].

In summary, we present, to our knowledge, the largest single-center observational study of DSD in newborns and CYP reported over a 25-year period. These data highlight the range of presentations, associated features, and approximate prevalence of these diverse conditions. This work should help in planning management strategies and policy going forward.

## Data Availability

Most quantitative data generated are presented in the accompanying tables, figures, and supplementary data sets [13]. Restrictions apply to the availability of some data generated or analyzed during this study to preserve patient confidentiality or because they were used under license. The corresponding author will on request detail the restrictions and any conditions under which access to some data may be provided.

## Abbreviations

21OHD: 21-hydroxylase deficiency
AIS: androgen insensitivity syndrome
BUDT: bilateral undescended testes
CAH: congenital adrenal hyperplasia
CAIS: complete androgen insensitivity syndrome
CYP: children and young people
DSD: differences (disorders) of sex development
FGR: fetal growth restriction
GD: gonadal dysgenesis
GOSH: Great Ormond Street Hospital
HH: hypogonadotropic hypogonadism
HSD: hydroxysteroid dehydrogenase
IUGR: intrauterine growth restriction
MDT: multidisciplinary team
MRKH: Mayer-Rokitansky-Küster-Hauser syndrome
ONS: Office for National Statistics
OTDSD: ovotesticular DSD
PMDS: persistent müllerian duct syndrome
PSH: penoscrotal hypospadias
SCDSD: sex chromosome DSD
SF-1/NR5A1: steroidogenic factor-1
STAR: steroidogenic acute regulatory protein/congenital lipoid adrenal hyperplasia
TDSD: testicular DSD
WT1: Wilms tumor 1
